# Clinical, Functional, and Radiological Evaluation of Pulmonary Involvement in Patients with Rheumatoid Arthritis

**DOI:** 10.3390/diagnostics16213669

**Published:** 2026-11-06

**Authors:** Neslihan Ozcelik, Elvan Senturk Topaloglu, Gül Devrimsel, Münevver Serdaroğlu Beyazal, Osman Cüre, Nur Hürsoy, Eray Halisdemir, Songul Ozyurt, Bilge Yilmaz Kara, Aziz Gumus

**Affiliations:** 1 Department of Pulmonology, Faculty of Medicine, Recep Tayyip Erdogan University, 53100 Rize, Turkey; neslihan.ozcelik@erdogan.edu.tr (N.O.); erayhalisdemir@live.com (E.H.); songul.ozyurt@erdogan.edu.tr (S.O.); aziz.gumus@erdogan.edu.tr (A.G.); 2 Department of Physical Medicine and Rehabilitation, Faculty of Medicine, Recep Tayyip Erdogan University, 53100 Rize, Turkeymunevver.serdaroglu@erdogan.edu.tr (M.S.B.); 3 Department of Rheumatology, Faculty of Medicine, Recep Tayyip Erdogan University, 53100 Rize, Turkey; osman.cure@erdogan.edu.tr; 4 Department of Radiology, Faculty of Medicine, Recep Tayyip Erdogan University, 53100 Rize, Turkey; nur.hursoy@erdogan.edu.tr; 5 Department of Pulmonology, Faculty of Medicine, TOBB University of Economics & Technology, 06560 Ankara, Turkey

**Keywords:** rheumatoid arthritis, pulmonary involvement, high-resolution computed tomography, uric acid, pulmonary function tests

## Abstract

**Background**: Pulmonary involvement is one of the most serious extra-articular manifestations of rheumatoid arthritis (RA), frequently remaining clinically silent until irreversible lung damage develops. This study aimed to determine the prevalence of high-resolution computed tomography (HRCT)-detected pulmonary involvement in patients with RA; compare the clinical, laboratory, and pulmonary function characteristics of patients with and without pulmonary involvement; and identify factors associated with pulmonary abnormalities. **Methods**: In this single-center retrospective cross-sectional study, 141 patients fulfilling the 2010 ACR/EULAR classification criteria for RA who had undergone thoracic HRCT were included. Clinical characteristics, laboratory findings, disease activity scores, pulmonary function tests, and HRCT findings were compared between patients with and without pulmonary involvement. Logistic regression analyses were performed to identify independent predictors of pulmonary involvement, and receiver operating characteristic (ROC) analysis was used to evaluate the diagnostic performance of serum uric acid. **Results**: HRCT identified pulmonary involvement in 33 of 141 patients (23.4%), with nonspecific interstitial pneumonia being the most common radiological pattern (39.4%), followed by usual interstitial pneumonia (24.2%). Patients with pulmonary involvement had significantly higher serum uric acid concentrations (5.65 ± 1.62 vs. 4.51 ± 1.31 mg/dL, *p* < 0.001), lower serum albumin levels (38.8 ± 4.9 vs. 40.9 ± 3.5 g/L, *p* = 0.008), higher platelet counts (*p* = 0.006), and lower forced vital capacity and predicted FEV_1_ values (*p* = 0.010 and *p* = 0.019, respectively). In multivariable logistic regression analysis, serum uric acid remained the only independent predictor of pulmonary involvement (odds ratio, 1.656; 95% confidence interval, 1.187–2.311; *p* = 0.003). ROC analysis demonstrated acceptable discriminatory performance for serum uric acid (AUC = 0.713), with an optimal cutoff value of >5 mg/dL yielding 68% sensitivity and 70% specificity. **Conclusions**: Nearly one-quarter of patients with RA demonstrated HRCT-detected pulmonary involvement despite having a similar prevalence of respiratory symptoms. Elevated serum uric acid was independently associated with pulmonary involvement and may represent an inexpensive, readily available biomarker to identify patients who may benefit from targeted pulmonary evaluation. These findings support the integration of simple laboratory markers with clinical assessment to facilitate earlier recognition of RA-associated pulmonary disease.

## 1. Introduction

Rheumatoid arthritis (RA) is a systemic autoimmune disease characterized by chronic synovial inflammation and progressive joint destruction. Although articular involvement is the hallmark of the disease, persistent systemic inflammation may affect multiple organ systems. Pulmonary involvement represents one of the most important extra-articular manifestations of RA and is a major contributor to disease-related morbidity and mortality [1,2]. RA-associated pulmonary disease encompasses a broad spectrum of clinical manifestations, including interstitial lung disease (ILD), airway disease, rheumatoid pulmonary nodules, and pleural involvement [1,2]. Also, different pulmonary compartments may be affected simultaneously in individual patients, further emphasizing the heterogeneous nature of RA-associated lung disease [3].

RA-associated ILD (RA-ILD) is the most common and clinically significant form of pulmonary involvement. Recent systematic reviews and meta-analyses have shown that the reported prevalence of RA-ILD varies considerably according to the diagnostic methods used and the characteristics of the study population; however, the overall prevalence is estimated to be approximately 18–20% [4]. Male sex, older age, cigarette smoking, longer disease duration, higher disease activity, positivity for rheumatoid factor (RF), and anti-cyclic citrullinated peptide (anti-CCP) antibodies have consistently been identified as the principal risk factors for the development of RA-ILD [5,6,7].

RA-ILD exhibits diverse radiologic and histopathologic patterns, most commonly usual interstitial pneumonia (UIP) and nonspecific interstitial pneumonia (NSIP) [1,6]. Disease progression may result in progressive pulmonary fibrosis, declining pulmonary function, and impaired health-related quality of life. Furthermore, patients with RA-ILD have a substantially higher risk of mortality than patients with RA who do not have pulmonary involvement [1].

Growing evidence indicates that pulmonary involvement frequently develops before the onset of respiratory symptoms. High-resolution computed tomography (HRCT) can detect interstitial lung abnormalities even in patients without dyspnea, cough, or exercise intolerance [6]. These findings suggest that pulmonary involvement in RA may remain subclinical for an extended period and that symptom-based assessment alone may fail to identify early disease.

HRCT is considered the most sensitive imaging modality for detecting RA-associated pulmonary involvement. However, because of its cost, radiation exposure, and limited accessibility, routine HRCT screening for all patients with RA is not feasible [1,6]. Although pulmonary function tests provide valuable information in the evaluation of pulmonary involvement, they may remain normal during the early stages of disease and lack sufficient diagnostic sensitivity when used in isolation [8,9]. Therefore, identifying patients at increased risk for pulmonary involvement and recognizing pulmonary disease at an early stage are of considerable clinical importance. Similarly, recent international expert consensus statements have emphasized the importance of early identification of RA-ILD and recommend implementing proactive screening strategies, particularly for patients with established risk factors [10].

Early detection of pulmonary involvement, followed by close clinical monitoring and timely initiation of appropriate therapeutic strategies, is critical for preventing the progression of fibrotic lung disease [1,6]. Nevertheless, data regarding the clinical characteristics of patients with HRCT-detected pulmonary involvement in the absence of overt respiratory symptoms and the laboratory parameters potentially associated with early pulmonary abnormalities remain limited.

The present study aimed to determine the prevalence of HRCT-detected pulmonary involvement in patients with RA; compare the clinical, functional, and laboratory characteristics of patients with and without pulmonary involvement; and identify factors associated with pulmonary involvement.

## 2. Materials and Methods

**Figure 1:**
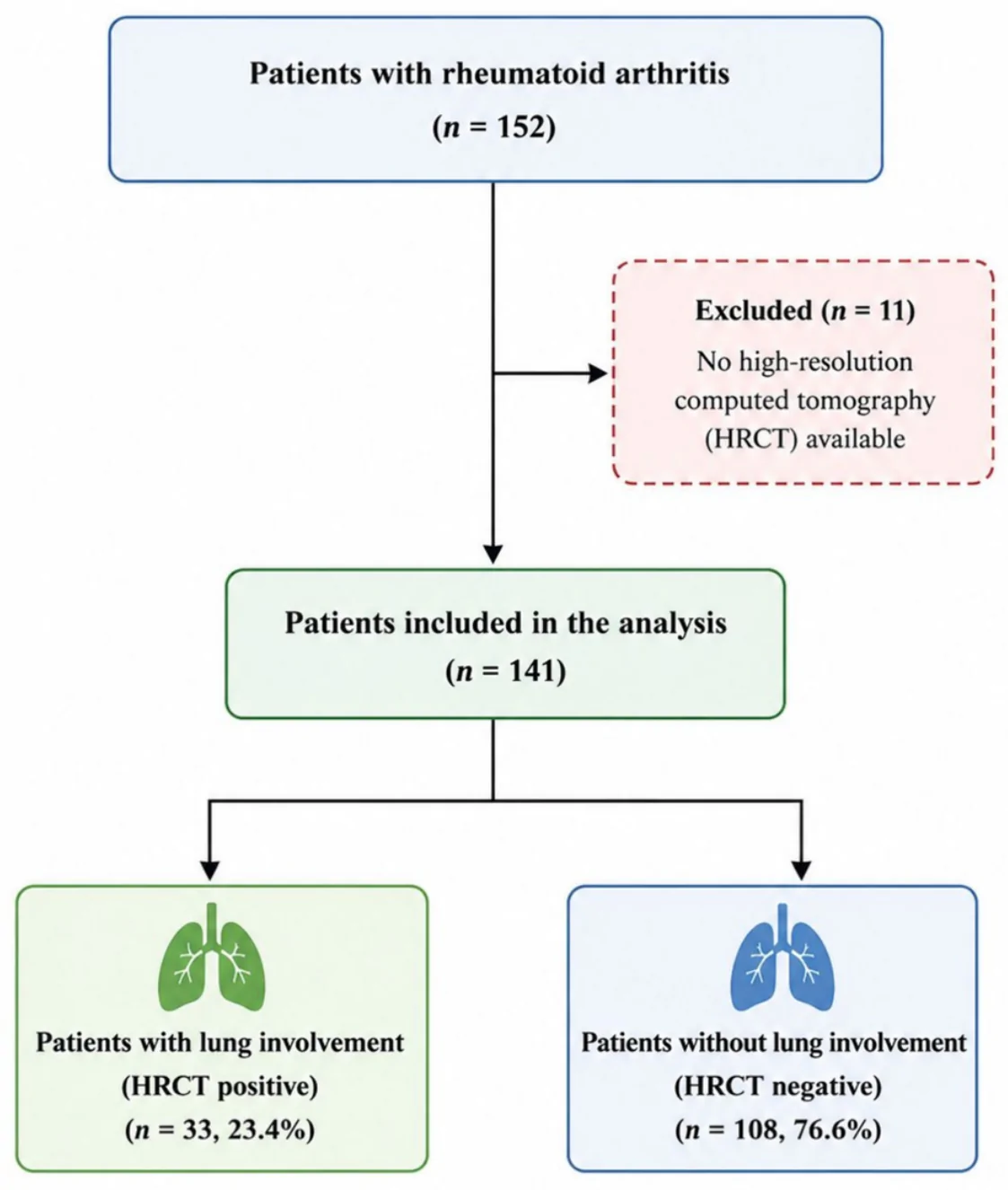
Flowchart of patient selection and study population.

This retrospective cross-sectional study was conducted through a review of the medical records of patients with RA who were followed at the Departments of Rheumatology, Physical Medicine and Rehabilitation, and Pulmonology of a tertiary care teaching and research hospital. Patients who fulfilled the 2010 American College of Rheumatology/European League Against Rheumatism (ACR/EULAR) classification criteria for RA and had undergone thoracic HRCT during follow-up were eligible for inclusion. Patients with concomitant systemic connective tissue diseases or overlap syndromes, such as systemic sclerosis or Sjögren’s syndrome, were not included in the study. Eleven patients without available thoracic HRCT examinations were excluded, leaving a total of 141 patients for the final analysis (Figure 1). The study protocol was approved by the Non-Interventional Clinical Research Ethics Committee of Recep Tayyip Erdoğan University (Approval No. 2024/307) and was conducted in accordance with the principles of the Declaration of Helsinki. The requirement for obtaining informed consent was waived by the Ethics Committee because of the retrospective nature of the study.

Data on age, sex, body mass index, smoking status, disease duration, comorbidities, current medications, disease activity scores (Disease Activity Score in 28 Joints and Clinical Disease Activity Index) and laboratory parameters [RF, anti-CCP, erythrocyte sedimentation rate (ESR), C-reactive protein (CRP), uric acid, albumin, and other routine biochemical parameters] were retrospectively obtained from the electronic medical record system.

Pulmonary involvement was determined based on the evaluation of thoracic HRCT images. All HRCT scans were independently reviewed by a thoracic radiologist and a pulmonologist, both with substantial experience in the assessment of thoracic imaging. Fibrotic changes consistent with ILD, reticular opacities, ground-glass opacities, rheumatoid pulmonary nodules, and bronchiectasis were considered radiologic evidence of pulmonary involvement. Patients with pulmonary abnormalities identified on HRCT were classified as the pulmonary involvement group, whereas those without radiologic evidence of pulmonary involvement were assigned to the non-pulmonary involvement group.

Available pulmonary function test results were reviewed. Forced vital capacity (FVC), forced expiratory volume in one second (FEV_1_), the FEV_1_/FVC ratio, and oxygen saturation were recorded and compared between the two groups.

All statistical analyses were performed using IBM SPSS Statistics version 27.0 (IBM Corp., Armonk, NY, USA). The distribution of continuous variables was assessed using the Kolmogorov–Smirnov test. Normally distributed variables were presented as mean ± standard deviation, whereas non-normally distributed variables were expressed as median (interquartile range [IQR]). Categorical variables were presented as frequencies and percentages. Between-group comparisons were performed using the independent-samples t test for normally distributed continuous variables and the Mann–Whitney U test for non-normally distributed variables. Categorical variables were compared using the chi-square test. Univariate and multivariate logistic regression analyses were performed to identify factors associated with pulmonary involvement. Variables significantly associated with pulmonary involvement in univariable logistic regression analysis were entered into the multivariable model. The results were reported as odds ratios (ORs) with corresponding 95% confidence intervals (CIs). The discriminatory performance of serum uric acid for predicting pulmonary involvement was evaluated using receiver operating characteristic (ROC) curve analysis. The area under the ROC curve (AUC), sensitivity, specificity, positive predictive value, and negative predictive value were calculated. All statistical tests were two-sided, and a *p*-value of <0.05 was considered statistically significant.

## 3. Results

During the study period, 152 patients with RA were assessed for eligibility. Eleven patients without available thoracic HRCT examinations were excluded, resulting in a final study cohort of 141 patients. The mean age was 63 ± 11 years, 116 patients (82.3%) were female, and the median disease duration was 5 years (IQR, 3–8 years).

Pulmonary involvement was identified on HRCT in 33 patients (23.4%). Among these patients, 13 (39.4%) exhibited a NSIP pattern, 8 (24.2%) had a UIP pattern, 11 (33.3%) had rheumatoid pulmonary nodules, and 1 (3.0%) had bronchiectasis.

Patients with and without pulmonary involvement were comparable with respect to age, sex, body mass index, smoking status, disease duration, disease activity measures, and treatment regimens (Table 1). However, among the evaluated comorbidities, diabetes mellitus was significantly more common in patients with pulmonary involvement than in those without pulmonary involvement (30% vs. 14%, *p* = 0.033).

**Figure 2:**
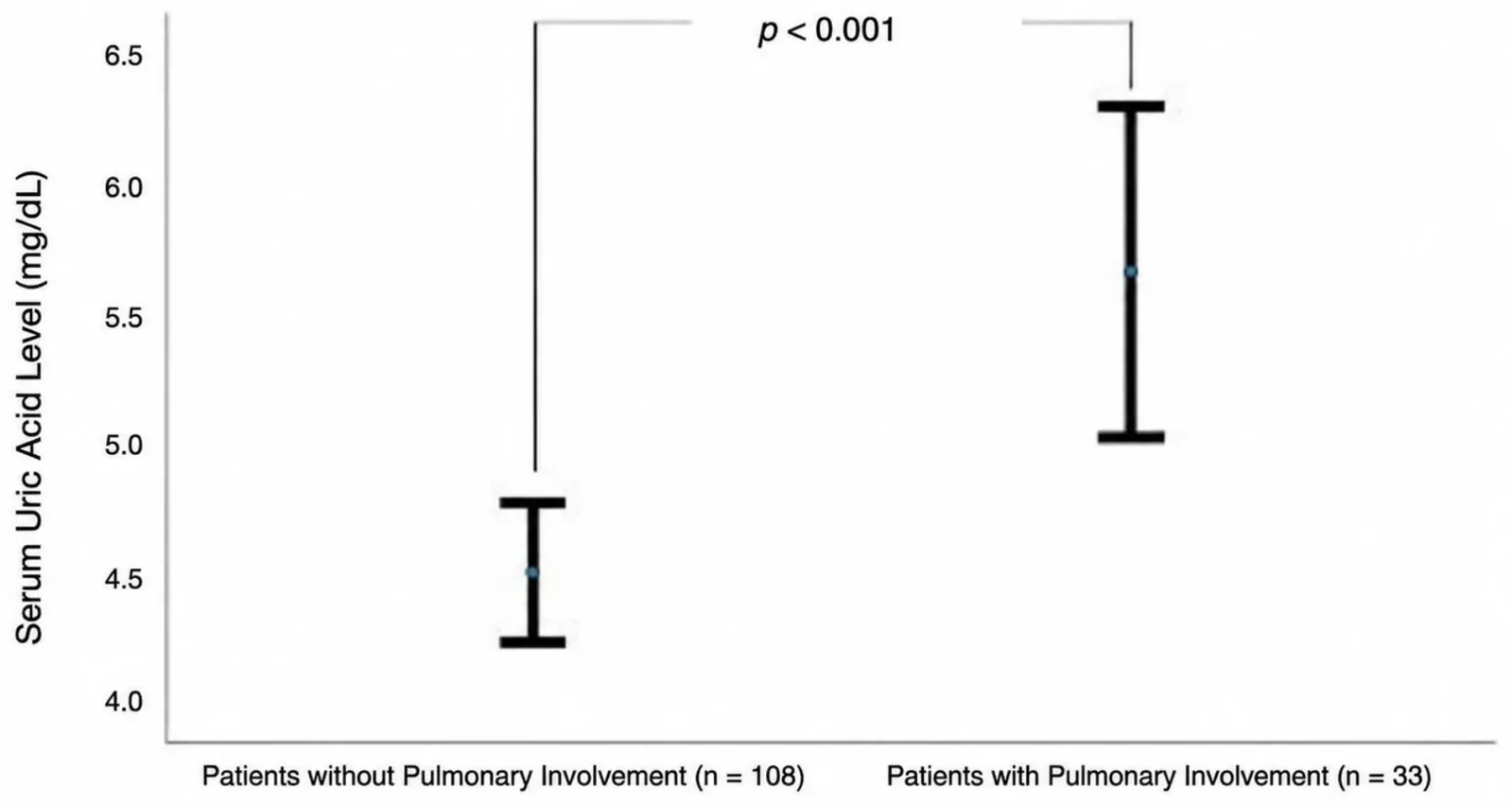
Comparison of serum uric acid levels between patients with RA with and without pulmonary involvement. RA, rheumatoid arthritis.

Patients with pulmonary involvement had significantly higher serum uric acid concentrations than those without pulmonary involvement (5.65 ± 1.62 vs. 4.51 ± 1.31 mg/dL, *p* < 0.001) (Figure 2). Conversely, serum albumin levels were significantly lower in the pulmonary involvement group (38.8 ± 4.9 vs. 40.9 ± 3.5 g/L, *p* = 0.008). Among inflammatory markers, CRP and ESR were both higher in patients with pulmonary involvement, whereas RF and anti-CCP antibody levels did not differ significantly between the groups. Platelet counts were significantly higher in patients with pulmonary involvement (324 ± 95 × 10^3^/µL vs. 276 ± 78 × 10^3^/µL, *p* = 0.006) (Table 1).

Pulmonary function test data were available for 121 of 141 patients (85.8%), including 30 patients with and 91 patients without pulmonary involvement. The prevalence of respiratory symptoms did not differ significantly between the two groups. The frequencies of chest pain, cough, and dyspnea were comparable. However, patients with pulmonary involvement had significantly lower predicted FVC and predicted FEV_1_ values than those without pulmonary involvement (*p* = 0.010 and *p* = 0.019, respectively) (Table 2).

In univariate logistic regression analysis, diabetes mellitus (OR, 2.667; 95% CI, 1.061–6.701; *p* = 0.037), lower serum albumin levels (OR, 0.875; 95% CI, 0.788–0.971; *p* = 0.012), higher serum uric acid levels (OR, 1.702; 95% CI, 1.244–2.329; *p* < 0.001), and higher platelet counts (OR, 1.007; 95% CI, 1.002–1.011; *p* = 0.008) were significantly associated with pulmonary involvement (Table 3).

In multivariate logistic regression analysis, elevated serum uric acid remained the only independent predictor of pulmonary involvement (OR, 1.656; 95% CI, 1.187–2.311; *p* = 0.003) (Table 4).

**Figure 3:**
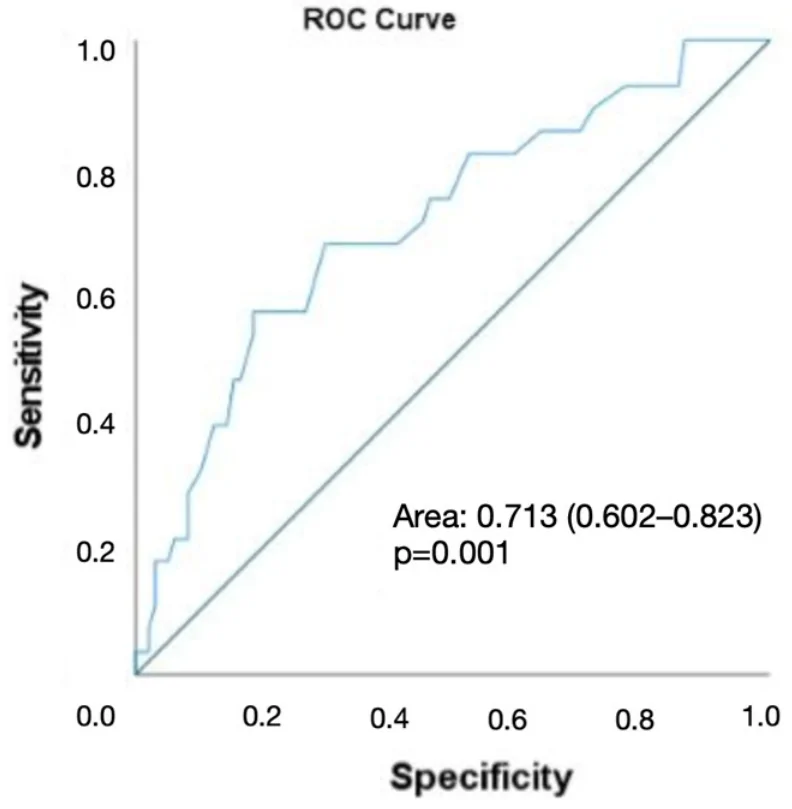
ROC curve showing the diagnostic performance of serum uric acid levels for identifying pulmonary involvement in patients with RA. ROC, receiver operating characteristic; RA, rheumatoid arthritis.

ROC curve analysis demonstrated that serum uric acid had acceptable discriminative performance for predicting pulmonary involvement (AUC, 0.713; 95% CI, 0.602–0.823; *p* = 0.001). The optimal cutoff value was >5 mg/dL, yielding a sensitivity of 68%, a specificity of 70%, a positive predictive value of 40%, and a negative predictive value of 88% (Figure 3).

## 4. Discussion

In this retrospective cross-sectional study, HRCT identified pulmonary involvement in 23.4% of patients with RA. Compared with patients without pulmonary involvement, those with pulmonary involvement exhibited a higher prevalence of diabetes mellitus, higher serum uric acid levels, lower serum albumin levels, and higher platelet counts. However, after adjustment for potential confounders, elevated serum uric acid remained the sole independent predictor of pulmonary involvement. In addition, patients with pulmonary involvement exhibited significantly lower FVC and percent predicted FEV_1_ values. Notably, despite a similar prevalence of respiratory symptoms in the two groups, HRCT detected clinically meaningful pulmonary abnormalities in patients with pulmonary involvement.

The prevalence of pulmonary involvement observed in the present study (23.4%) was consistent with contemporary evidence. In a systematic review and meta-analysis by Wang et al. [4] that included 56 studies comprising 11,851 patients with RA-ILD, the pooled prevalence of RA-ILD was estimated at 18.7%. Similarly, more recent global meta-analyses have reported that, although prevalence estimates vary according to the diagnostic modality employed, most cohorts report rates ranging from 15% to 25% [11]. In contrast to previous reports indicating UIP as the predominant radiological pattern in RA-associated ILD [12], NSIP was more frequent than UIP in our cohort. This difference may be related to the relatively small number of patients with ILD, and the distribution of radiological patterns should therefore be interpreted with caution.

Pulmonary involvement in RA may remain clinically silent for a prolonged period, with radiologic abnormalities preceding the onset of overt respiratory manifestations. Recent prospective evidence from the SAIL-RA cohort further demonstrated that ILD may already be detectable by HRCT in patients with early RA, supporting the need for risk-based screening strategies rather than reliance on respiratory symptoms alone [13]. Abdelwahab et al. [8] demonstrated a substantial burden of subclinical ILD detected by HRCT among asymptomatic patients with RA and further showed that normal pulmonary function test results were insufficient to exclude pulmonary involvement. More recent review articles and international expert consensus statements have consistently emphasized that clinical manifestations are frequently absent during the early stages of RA-ILD and have advocated proactive screening strategies, particularly for individuals with established risk factors [13,14,15,16]. Consistent with these observations, the frequencies of dyspnea, cough, and chest pain were comparable between patients with and without pulmonary involvement in our cohort, despite the presence of significant pulmonary abnormalities on HRCT. Collectively, these findings indicate that reliance on symptom-based assessment alone is likely to result in underrecognition of early pulmonary involvement.

The significantly lower FVC and percent predicted FEV_1_ values observed in patients with pulmonary involvement further underscore the functional consequences of pulmonary disease in RA. Recent real-world data similarly indicate that pulmonary function testing, particularly when DLCO is incorporated with spirometry and clinical risk factors, may improve the identification of patients with HRCT-confirmed RA-ILD [9]. Although respiratory symptoms were not more frequent in these patients, objective impairment in pulmonary function was already evident. Restrictive ventilatory dysfunction and reduced diffusing capacity represent the most characteristic physiological abnormalities in RA-ILD and are well recognized to precede the development of clinically apparent respiratory disease [1,17]. Therefore, our findings reinforce the concept that pulmonary function may be compromised even in asymptomatic individuals and that clinically relevant pulmonary involvement may remain undetected if evaluation is based exclusively on symptoms.

The results also showed that diabetes mellitus was more prevalent among patients with pulmonary involvement. Although diabetes mellitus was associated with pulmonary involvement in the univariate analysis, this association was no longer significant in the multivariate analysis. The relationship between diabetes mellitus and ILD has attracted increasing attention in recent years. Hyperglycemia-induced oxidative stress, microvascular injury, and persistent low-grade inflammation have all been proposed as mechanisms that may contribute to pulmonary fibrogenesis [4]. However, the role of diabetes mellitus in the pathogenesis of RA-ILD remains inadequately understood, and the available evidence remains inconclusive.

Another noteworthy finding of the present study was the lower serum albumin levels observed in patients with pulmonary involvement. Serum albumin is widely regarded as an indirect marker of systemic inflammation, and hypoalbuminemia has consistently been associated with adverse clinical outcomes in chronic inflammatory diseases [18]. In patients with RA-ILD, lower serum albumin levels may reflect a greater inflammatory burden and more advanced pulmonary injury. Although serum albumin was significantly associated with pulmonary involvement in the univariate analysis, it did not remain an independent predictor in the multivariate analysis. This finding suggests that serum albumin is more likely to represent a surrogate marker of the overall inflammatory state rather than a direct contributor to pulmonary involvement.

Recent evidence also suggests that serological profiles beyond conventional RF and anti-CCP antibodies may contribute to pulmonary risk stratification in RA. In the SETOUCHI-RA registry, Nakano et al. reported that specific ANA staining patterns, particularly the nucleolar pattern, were independently associated with pulmonary complications, predominantly ILD, highlighting the potential value of readily available laboratory markers in identifying patients at increased pulmonary risk [19].

The role of serum uric acid in RA-ILD has attracted increasing attention in recent years. Wang et al. [20] demonstrated significantly higher serum uric acid levels in patients with RA-ILD and proposed serum uric acid as a potential diagnostic biomarker for pulmonary involvement. More recently, Hu et al. [21] reported that elevated serum uric acid levels were associated not only with pulmonary involvement but also with greater disease severity and poorer clinical outcomes. Collectively, these findings suggest that serum uric acid may reflect inflammatory and fibrotic processes beyond its conventional role as a metabolic marker [22]. However, because our endpoint included pulmonary abnormalities beyond ILD, the present findings should be interpreted as associations with overall HRCT-detected pulmonary involvement rather than specifically with RA-ILD.

Although our findings were generally consistent with the existing literature, they also provide several novel observations. While most previous studies have focused on patients with established RA-ILD, our study evaluated pulmonary involvement in patients with RA who underwent HRCT, most of whom were free of respiratory symptoms. Recent externally validated prediction models further support the feasibility of combining routinely available clinical and laboratory parameters to identify patients with RA who are at increased risk of ILD [23]. A recent systematic review and meta-analysis also concluded that circulating biomarkers and biomarker-based prediction models should currently be regarded as adjunctive tools for risk stratification and selection of patients for confirmatory HRCT rather than as replacements for imaging [24]. Within this context, serum uric acid levels were significantly higher in patients with pulmonary involvement and remained independently associated with pulmonary involvement in the multivariate analysis. In addition, the finding that a serum uric acid threshold of >5 mg/dL demonstrated acceptable diagnostic performance for predicting pulmonary involvement suggests that this readily available biomarker may facilitate the identification of patients at increased risk before the onset of respiratory symptoms. Therefore, given its widespread availability and low cost, serum uric acid may serve as a simple adjunctive marker that could help identify patients who may warrant more comprehensive pulmonary evaluation.

### Limitations

This study has several limitations. First, its retrospective, single-center design may limit the generalizability of the findings. Second, the relatively small sample size, particularly the limited number of patients with pulmonary involvement, precluded detailed subgroup analyses according to specific patterns of pulmonary involvement. Because inclusion was restricted to patients who had undergone HRCT, selection bias related to the clinical indication for imaging cannot be excluded. In addition, the pulmonary involvement endpoint comprised heterogeneous HRCT abnormalities and should therefore not be interpreted as being specific to RA-associated interstitial lung disease. The relatively limited number of pulmonary involvement events may also have affected the stability of the multivariable estimates. Third, several pulmonary function parameters that are important for the assessment of ILD, including diffusing capacity for carbon monoxide, were not available for all patients. As serum uric acid may be influenced by metabolic and renal factors not fully captured in this retrospective dataset, residual confounding cannot be excluded. Because of the cross-sectional design, the temporal and potentially causal relationship between serum uric acid levels and pulmonary involvement could not be established, and the impact of elevated serum uric acid on long-term clinical outcomes could not be evaluated. Consequently, our findings warrant further validation via larger, multicenter, prospective studies.

## 5. Conclusions

Pulmonary involvement is a common extra-articular manifestation of RA and may be detected before the development of clinically apparent respiratory symptoms. In the present study, approximately one-quarter of patients who underwent HRCT demonstrated evidence of pulmonary involvement, and elevated serum uric acid emerged as an independent predictor of pulmonary involvement. These findings suggest that serum uric acid, an inexpensive and readily accessible laboratory parameter, may serve as a potential adjunctive marker for identifying patients with RA who are at increased risk for pulmonary involvement in routine clinical practice. Nevertheless, larger multicenter prospective studies are warranted to validate these findings and to further establish the clinical utility of serum uric acid as a biomarker for pulmonary involvement in patients with RA.

## Figures and Tables

**Table 1:** Clinical and laboratory characteristics of patients with RA according to pulmonary involvement.

	Pulmonary Involvement (*n*: 33)	No Pulmonary Involvement (*n*: 108)	*p*-Value
Age, years, mean ± SD	64.9 ± 11.4	62.3 ± 10.9	0.233
Sex, female, *n* (%)	24 (73%)	92 (85%)	0.101
BMI (kg/m^2^) *	29.6 ± 5.5	30.5 ± 6.0	0.488
Smoking (current or former), *n* (%)	9 (27%)	19 (18%)	0.329
Smoking exposure (pack-years)	20 (17–30)	20 (15–30)	0.901
Disease duration (years)	5 (3–8)	5 (3–8)	0.966
Comorbidities			
≥1 comorbidity	23 (70%)	65 (60%)	0.352
CAD	4 (12%)	24 (22%)	0.131
Diabetes mellitus (DM)	10 (30%)	15 (14%)	0.033
Hypertension (HT)	19 (57%)	49 (45%)	0.295
Asthma	6 (18%)	23 (21%)	0.389
Treatments			
Conventional therapy	19 (57%)	82 (76%)	0.121
Biologic therapy	10 (30%)	24 (22%)	0.232
Steroids	16 (48%)	58 (54%)	0.823
DAS-28 score *	3.54 ± 1.39	3.72 ± 1.39	0.544
CDAI *	9.5 ± 4.0	8.6 ± 4.7	0.377
RF (IU/mL)	87(53–135)	44 (16–118)	0.097
Anti-CCP (U/mL)	120 (4–362)	68 (3–199)	0.410
25(OH) vitamin D (ng/mL)	20 (9–24)	18(11–23)	0.899
Creatinine (mg/dL)	0.70 (0.62–0.89)	0.71 (0.63–0.81)	0.687
Uric acid (mg/dL)	5.65 ± 1.62	4.51 ± 1.31	<0.001
Total Protein (g/L)	71 ± 8	72 ± 6	0.718
Albumin (g/L)	38.8 ± 4.9	40.9 ± 3.5	0.008
AST (U/L)	19 (16–26)	19 (17–21)	0.321
ALT (U/L)	16 (12–23)	17 (13–27)	0.282
LDH (U/L)	190 (163–231)	199 (165–227)	0.773
CRP (mg/L)	8.6 (4.1–23.9)	4.8 (2.2–12.9)	0.035
Hb (g/dL)	12.6 ± 1.3	12.7 ± 1.2	0.727
Erythrocyte sedimentation rate (mm/hour)	24 (17–35)	17 (10–31)	0.034
Leukocyte (/µL)	7982 ± 2520	7671 ± 2481	0.538
Neutrophil (/µL)	4946 ± 2200	4701 ± 1866	0.534
Lymphocyte (/µL)	2278 ± 886	2190 ± 794	0.596
NLR	1.81 (1.47–2.58)	2.03 (1.55–2.71)	0.569
Platelets (×10^3^/µL)	324 ± 95	276 ± 78	0.006
MPV (fL)	10.2 ± 1.1	10.5 ± 1.4	0.176
RDW-CV (%)	14.5 ± 1.2	14.6 ± 1.4	0.881

Data are presented as mean ± standard deviation, median (interquartile range, 25–75%), or *n* (%), as appropriate. * RA: rheumatoid arthritis; BMI: body mass index; DAS-28: Disease Activity Score-28; CDAI: Clinical Disease Activity Index; RF: rheumatoid factor; Anti-CCP: anti-cyclic citrullinated peptide antibody; CRP: C-reactive protein; AST: aspartate aminotransferase; ALT: alanine aminotransferase; LDH: lactate dehydrogenase; NLR: neutrophil/lymphocyte ratio; MPV: mean platelet volume; RDW-CV: red blood cell distribution width-coefficient of variation; SD: standard deviation.

**Table 2:** Comparison of respiratory symptoms and pulmonary function test parameters between patients with RA with and without pulmonary involvement.

	Pulmonary Involvement (*n*: 33)	No Pulmonary Involvement (*n*: 108)	*p*-Value
Respiratory symptoms			
Chest pain	4 (12%)	13 (12%)	0.589
Cough	8 (24%)	16 (15%)	0.286
Dyspnea	10 (30%)	28 (26%)	0.775
FVC (mL)	2336 ± 989	2581 ± 662	0.131
FVC, (%) predicted	82 ± 18	91 ± 17	0.010
FEV_1_ (mL)	2102 ± 891	2326 ± 582	0.122
FEV_1_, (%) predicted	91 ± 22	100 ± 18	0.019
FEV_1_/FVC	90 ± 8	90 ± 7	0.115
Oxygen saturation (%)	96.1 ± 4.3	97.4 ± 1.6	0.148

RA: rheumatoid arthritis; FVC: forced vital capacity; FEV_1_: forced expiratory volume in one second.

**Table 3:** Univariate logistic regression analysis of factors associated with pulmonary involvement in patients with RA.

Variables	OR	95% Confidence Interval	*p*-Value
DM	2.667	1.061–6.701	0.037
Albumin (g/L)	0.875	0.788–0.971	0.012
Uric acid (mg/dL)	1.702	1.244–2.329	<0.001
Erythrocyte sedimentation rate (mm/hour)	1.017	0.994–1.039	0.142
CRP (mg/L)	1.006	0.996–1.015	0.243
Platelets (×10^3^/µL)	1.007	1.002–1.011	0.008

RA: rheumatoid arthritis; OR: odds ratio; DM: diabetes mellitus; CRP: C-reactive protein.

**Table 4:** Multivariate logistic regression analysis of factors independently associated with pulmonary involvement in patients with RA.

Variables	OR	95% Confidence Interval	*p*-Value
DM	1.960	0.616–6.236	0.255
Albumin (g/L)	0.921	0.807–1.050	0.218
Uric acid (mg/dL)	1.656	1.187–2.311	0.003
Platelets (×10^3^/µL)	1.002	0.996–1.008	0.479

RA: rheumatoid arthritis; OR: odds ratio; DM: diabetes mellitus.

## Data Availability

All data generated or analyzed during this study are included in this article. The data will be available upon reasonable request (contact: elvan.senturktopaloglu@erdogan.edu.tr).
